# Not uni-dimension! Exploring the multi-dimensional concept of positive body image in the Chinese context

**DOI:** 10.3389/fpsyg.2025.1494172

**Published:** 2025-01-21

**Authors:** Hanqin Yan

**Affiliations:** School of Public Administration, Hohai University, Nanjing, China

**Keywords:** Chinese college students, positive body image, measurement invariance, latent profile analysis, validation

## Abstract

**Objectives:**

Past researchers often used single measurement to explore the overall level of positive body image, which caused much misunderstanding of positive body image. With a positive psychology perspective, and based on the holistic positive body image theory, this study explored the multi-dimensional concept of positive body image in the Chinese context, integrated its three measures, examined the overall level of positive body image among Chinese college students, and tested the gender measurement invariance, as well as explored the latent categories of positive body image in the college student population.

**Methods:**

A convenience sampling method was used to survey 670 college students in China, using the Chinese version of Body Appreciation Scale-2 (BAS-2), Functionality Appreciation Scale (FAS), Body Image Acceptance and Action Questionnaire-5 (BI-AAQ-5), and the Positive Body Talk Scale (PBTS). Confirmatory factor analysis was used to validate the three dimensions of positive body image, and a measurement invariance test was used to figure out the measurement invariance of the three scales on the gender variable, lastly, latent profile analysis was used to explore the latent categories of positive body image among Chinese college students.

**Results:**

(1) Three measures of positive body image were able to measure Chinese college students' overall positive body image well, and had good measurement invariance on the variable of gender; (2) Chinese college students' positive body image could be divided into three potential categories, namely, “low-level of positive body image” (32.2%), “general-level positive body image” (40.4%), and “high-level positive body image” (27.4%); (3) positive body talk can significantly and positively predict the overall positive body image of Chinese college students.

**Conclusion:**

We conclude that the three measures of positive body image are valid and versatile to Chinese college students and have high relevance with positive body talk, we also validate the multidimensional concept of positive body image in the Chinese context and hope to lead future Chinese researchers for better understanding the multi-dimensional concept of positive body image, and use the three measures to measure the holistic positive body image. We reveal and identify three latent categories of positive body image to effectively promote the healthy development of Chinese college students' macro-ecosystem.

## 1 Introduction

Since the twenty-first century, body image problems (e.g., body dissatisfaction) and their resulting eating disorder pathology have been prevalent in Chinese populations, ranging from children to older adults (Chen and Jackson, 2008; Tong et al., 2014; Wang et al., 2018; Liu et al., 2019; Li et al., 2022; Zhang et al., 2023), and body image disorders in college student populations have continued to climb (Wu et al., 2022). The college population is at an important stage in the transition from adolescence to young adulthood and is at a essential time in the development of body image, self-identity, and social homogeneity (Gattario and Frisén, 2019), and it is critical to promote positive body image in the college population, rather than seeking relief after body dissatisfaction, to improve their overall mental health (Webb et al., 2015).

Body Image refers to an individual's thoughts, perceptions, and behaviors about his or her body (Thompson et al., 1999), divided into Negative Body Image and Positive Body Image. Negative body image refers to individuals' negative evaluations, dissatisfaction, or shame about their bodies (Gattario et al., 2020). In the past, research on body image has focused on negative body image (Grogan, 2021), with a particular focus on the mechanisms by which negative body image affects an individual's physical and mental health, which severely impacts an individual's psychological wellbeing, leading to low self-efficacy, low self-worth, depression and eating disorders (Murray et al., 2019). With the rise of positive psychology, scholars in China have begun to turn their attention to positive body image (Chen et al., 2020; Liu and Zhou, 2022; Yang et al., 2023), asserting that positive and negative body image are somehow independent of each other, rather than different ends of the same continuum. If body image therapies alleviate the symptoms of negative body image but do not enhance aspects of positive body image, they may at best promote a neutral body image (Tylka and Wood-Barcalow, 2015a). Positive body image has a protective role across a range of mental and physical health outcomes and therefore deserves more research attention (Gillen, 2015).

Tylka and Wood-Barcalow (2015a) note that positive body image is not a uni-dimensional conceptual structure, but a multidimensional one that primarily includes attributes such as body appreciation, functionality appreciation, and filtering information in a way that protects the body (Tylka and Wood-Barcalow, 2015a), is related to various aspects of an individual's physical and mental health and health behaviors, such as intuitive eating (Chen et al., 2020), and is an important entry point for exploring the health of an individual's development. Additionally, positive body image has been linked to self-esteem, life satisfaction, and self-compassion, and is thought to protect individuals from appearance-related messages and pressures to conform to sociocultural appearance ideals (Andrew et al., 2016; Tylka and Wood-Barcalow, 2015a). Positive body image has an internal and external effect on individuals, favoring the healthy development of their macro-ecosystems (Yang et al., 2023). Positive psychology points out that positive character traits may not simply represent the absence of negative character traits, for example, fostering positive affect is more clinically therapeutically effective than simply reducing negative affect (Fredrickson and Losada, 2005).

As a core component of positive body image, Body Appreciation refers to individuals maintaining a positive and favorable attitude toward the appearance of their body (e.g., body shape, appearance, etc.), respecting and pleasing their own body (Linardon et al., 2022). To date, body appreciation has been one of the most researched aspects of positive body image, measured through the Body Appreciation Scale-2 (Tylka and Wood-Barcalow, 2015b). As a significant predictor of an individual's mental health, body appreciation significantly predicts an individual's mental health 1 year later (Urke et al., 2021). At the same time, body appreciation has a high protective potential against eating disorder symptoms in adult women, predicting a lower likelihood of eating disorder emergence, and thus promises to be a viable target for intervention in mental health promotion initiatives and eating disorder prevention programs (Linardon et al., 2022). The idea that body appreciation is also a central construct of the intuitive eating acceptance model (Avalos and Tylka, 2006) is a good explanation of how positive environmental influences can promote intuitive eating in individuals through body appreciation. Meanwhile, a study showed that female college students who found dancing possessed a higher level of body appreciation than those who had never danced (Tiggemann, 2015). Also, researchers have found that a key element of sustained physical activity is recognizing that the body will in turn undergo positive changes over time, ultimately improving its positive body image (Nam et al., 2023a).

With the boom in research on positive body image by foreign scholars, Functionality Appreciation has been identified as the second important aspect of positive body image (Linardon et al., 2023), which refers to an individual's ability to appreciate, honor, and respect the body and involves functional, bodily abilities related to the body's internal processes, senses and feelings, creative activities, communication with others, and self-care, not just awareness of body functions (Alleva et al., 2017). However, most of the current national research on positive body image has focused on individual body appreciation (Ma et al., 2020; Gao et al., 2022; Yan et al., 2024), while neglecting functionality appreciation. Indeed, the lack of research incorporating functionality appreciation has been identified as one of the key limitations in the field of body image (Smolak and Cash, 2011). Therefore, noting gratitude for what the body can do or is capable of doing, rather than just what it can do or is capable of doing, is more in line with the definition of positive body image (Alleva et al., 2015; Tylka and Wood-Barcalow, 2015a). In addition to exploring an individual's body appreciation, examining an individual's appreciation for body functioning is an important way to obtain a more complete and comprehensive positive body image of an individual (Alleva and Tylka, 2021). Alleva et al. (2015) demonstrated through an experimental study that functionality appreciation was effective in reducing an individual's body dissatisfaction and preoccupation with appearances, which in turn led to a more positive body image; and Zeng et al. (2021) found after conducting an experiment with a group of female college students that interventions for writing about body functioning were effective in increasing individuals' functionality appreciation, which in turn lowered their levels of negative body image. At the same time, participation in sports helped adolescent boys and girls from Sweden to focus on and appreciate the functionality of their bodies, and their positive view of their bodies promoted their reasons for participating in sports related to self-care, enjoyment, and challenge (Frisén and Holmqvist, 2010). A meta-analysis study showed that the most influential motivation driving individuals to participate in sport was the perceived benefits in terms of physical functionality, and that this effect was more pronounced in female individuals, who did not engage in sport primarily out of interest or enjoyment, but rather were more focused on considerations related to physical functioning, with females showing more concern than males (Nam et al., 2023b).

Meanwhile, what has been identified as the third important aspect of positive body image is Body Image Flexibility (Alleva et al., 2017; Linardon et al., 2021). Body Image Flexibility is a concept derived from psychological flexibility, which refers to an individual's ability to be fully aware of and experience the ongoing feelings, sensations, thoughts, and beliefs of his or her body and to act on them according to his or her values (Sandoz et al., 2013), and it has attracted a great deal of attention from national and international scholars in recent years. A growing body of empirical research has revealed the potentially central role of body image flexibility in the prevention and treatment of eating disorders (Rogers et al., 2018; Pellizzer et al., 2018), being positively associated with intuitive eating and physical and mental health, and significantly negatively associated with body dissatisfaction and eating disorders (Soulliard and Vander Wal, 2019), is a mediator and moderator of the relationship between body dissatisfaction and eating disorders (Sandoz et al., 2013; Timko et al., 2014).

Although there have been many previous studies on positive body image, there are still several areas that need to be clarified. First, Chinese scholars have basically used the uni-dimensional Body Appreciation Scale-2 to test the measurement of positive body image (Tang et al., 2024; Jia and Chen, 2024), and Yang et al. (2023) pointed out in their study that the uni-dimensional Body Appreciation Scale-2, along with multidimensional theoretical constructions (Tylka and Wood-Barcalow, 2015b) and the operational definition of positive body image (Chen et al., 2015) still have some structural differences, and suggested that future research should add to improve the measures of positive body image to deepen people's understanding of its connotation and structure. At the same time, most of the entry statements of this scale only probe about the external aspects of the body, such as body shape (“I have a positive attitude toward my body shape”) and body size (“I appreciate the unique and different body shapes I have”), and do not allow for an in-depth examination of individuals' on other aspects of positive body image, such as functionality appreciation, and body image flexibility. Functionality appreciation, and body image flexibility have also been shown to differ from other aspects of positive body image (Alleva et al., 2017; Swami et al., 2020). Thus, this study combines the three measures of body appreciation, functionality appreciation, and body image flexibility to comprehensively examine the level of positive body image among Chinese college students.

Second, gender plays a unique role in the formation of body image (Morrison and McCutcheon, 2012), and cross-cultural studies have found that males have more positive body image than females (Casale et al., 2021; Ma et al., 2020; Swami et al., 2021), however, this discrepancy could arise from inherent psychological differences between genders in different cultures, or from the variance of the questionnaire itself. Ensuring measurement invariance of scales is a prerequisite for valid cross-group comparisons (Riordan and Vandenberg, 1994). To ensure that cross-group gender comparisons are meaningful, it is firstly necessary to ensure that the questionnaire has cross-gender invariance. There is currently no research in the country that has examined the measurement invariance of the positive body image measures on the gender variable especially in the Chinese context. Therefore, one of the aims of the present study was to examine whether the three measures assessing positive body image had cross-gender measurement invariance.

Secondly, previous studies have explored more about the influence mechanism of positive body image, but most of them have adopted a variable-centered approach, focusing only on sample averages and ignoring the heterogeneity within the group and its internal connections. The latent profile analysis method is a subject-centered analysis method that can effectively compensate for this shortcoming by identifying differentiated effector characteristics from the subjects' own characteristics (Spurk et al., 2020). Therefore, it is necessary to adopt the latent profile analysis method to incorporate the categories of the three aspects of positive body image at the same time, and to analyse their latent types and their internal relationships from a holistic perspective.

Finally, the influencing factors of positive body image are yet to be further explored. As a multidimensional concept, the influencing factors of positive body image are complex. Based on the biopsychosocial model proposed by Ricciardelli et al. (2003), it is argued that biological, psychological, and social factors combine to influence the formation and change of positive body image in individuals. Past research has examined relatively few social factors and lacks quantitative indicators. One study suggests that positive body talk with peers is beneficial to individuals' body esteem and body satisfaction (Rudiger and Winstead, 2013). Body Talk, a significant risk factor for body dissatisfaction and eating disorders (Cruwys et al., 2016), has a significant impact on an individual's body image, and refers to an individual's disclosure of thoughts, emotions, or attitudes about his or her own body in conversations with others, which can be categorized into negative body talk (i.e., talking about one's own body in a demeaning way) and positive body talk (talking about one's body in an accepting and appreciative way) (Hart and Chow, 2020). However, current research on body talk by scholars in China is limited to negative body talk, especially fat talk among women. With the boom in positive psychology research, scholars in the field have begun to turn their attention to how positive emotions and behaviors can contribute to an individual's psychological wellbeing, and thus, the present study focuses attention on the effects of positive body talk behaviors on an individual's positive body image.

In the present study, we aim to integrate the three measurement tools of positive body image to examine the level of positive body image among college students as a whole, and to test whether the measurement tools of positive body image have measurement invariance on the variable of gender in the college student population, and finally to examine the potential categories of positive body image and their predictors in the college student population, in order to further clarify the mechanism of the influence of positive body image. The final study examines the potential categories of positive body image and their predictors, providing a reference for further clarifying the mechanisms of positive body image.

## 2 Method

### 2.1 Participants

The study adopts the convenience sampling method, we selected four university students in Nanjing City, Jiangsu Province, China to fill out the questionnaire. College students are from Hohai University, Nanjing Normal University, Southeast University, and Nanjing University. A total of 670 questionnaires were recovered, and 625 valid questionnaires were obtained through lie detector questions, after excluding questionnaires with too short or too long response time, the effective recovery rate was 93.28%. The mean age of valid subjects was 20.14 years (*SD* = 1.92). See the demographic details in Table 1.

**Table 1:** Descriptive statistics of demographic variables.

**Variable**	**Option**	**Number**	**%**
Sex	Male	399	63.84%
	Female	226	36.16%
Educational level	Undergraduate	423	67.68%
	Postgraduate	202	32.32%
Major	Humanities	258	41.28%
	Sciences	255	40.80%
	Sports	112	17.92%

### 2.2 Procedures

We first obtained the ethical approval for the study from the Ethics Committee of the Psychology Department of the first author's university (Hohai University) in December 2023 (Approval Number: 2023120401). The privacy and anonymity of the study were emphasized, we wrote a confidentiality agreement regarding personal information and collected and organized the scales used to finalize the questionnaire for the actual administration of the questionnaire. Finally, the distribution of the questionnaire was in January 2024 and took place at the first author's university. We asked for an available office room so that college students could finish the questionnaire without disturbance.

Guidelines for the questionnaire are labeled at the top of the page, “Dear students, Hello! We are researchers from the Institute of Psychology and we are currently working on a project about college students' body image. This questionnaire may take up your valuable 5-min time to fill in, only for the exploration of college students' body image research, anonymous form of filling in, the results of the confidentiality of treatment, will not disclose your personal information! All the questions in the questionnaire are not right or wrong, please choose the most suitable option according to your personal situation, thank you very much for your support”. Participants were asked to complete a list of questions on demographic items (age and education level), body appreciation, functionality appreciation, body image flexibility and positive body talk scale in sequential order. Finally, the consent was obtained from all participants prior to data collection. In February 2024, all questionnaire data was entered into SPSS. Then we finished data collection and began the data analysis in March 2024.

### 2.3 Measures

#### 2.3.1 Body appreciation

The Body Appreciation Scale-2 (BAS-2; Tylka et al., 2022) was used to measure the body appreciation level. The scale is scored on a 5-point Likert scale, with 1 meaning “never” and 5 meaning “always”, and the items are positively scored. Higher mean scores on the scale indicate higher levels of body appreciation. In this study, the scale had a Cronbach's α coefficient of 0.90, a good fit for validity indicators, χ^2^/*df* = 1.27, RMSEA = 0.01, CFI = 0.99, TLI = 0.99, and SRMR = 0.02.

#### 2.3.2 Functionality appreciation

The study used the Chinese version of Functionality Appreciation Scale (FAS; Alleva et al., 2017; He et al., 2023a) to measure the extent to which individuals appreciated their body functions. The scale consists of seven items with questions such as “I feel good about my body”. The scale is scored on a 5-point Likert scale, with 1 being “strongly disagree” and 5 being “strongly agree”. The scale items are positively scored, with higher total scores indicating a higher level of appreciation of one's bodily functions. In this study, the scale had a Cronbach's α coefficient of 0.91, a good fit for validity indicators, χ^2^/*df* = 4.91, RMSEA = 0.06, CFI = 0.97, TLI = 0.96, and SRMR = 0.02.

#### 2.3.3 Body image flexibility

The Chinese version of Body Image Acceptance and Action Questionnaire-5 (BI-AAQ-5; Sandoz et al., 2013; He et al., 2021) was used to assess the flexibility of individuals' body image. The scale consists of five items, such as “worrying about my weight makes it difficult for me to live a life that I find rewarding”. The scale is scored on a 7-point scale where 1 is “never” and 7 is “always”. The scale items are reverse-scored to assess an individual's body image flexibility, with higher total scores indicating higher body image flexibility. In this study, the scale had a Cronbach's α coefficient of 0.88, a good fit for validity indicators, χ^2^/*df* = 1.94, RMSEA = 0.03, CFI = 0.99, TLI = 0.99, and SRMR = 0.01.

#### 2.3.4 Positive body talk

The study used the Chinese version of Positive Body Talk subscale of the Body Talk Scale (Lin et al., 2021; He et al., 2023b) to measure the frequency with which individuals talk positively about their bodies when they talk to others in their daily lives. The scale consists of five items with questions such as “I feel good about my body”. The scale is scored on a 6-point scale which 1 is “never” and 6 is “always”. The scale items are positively scored, with a higher total score representing a higher frequency of positive body talk. In this study, the scale had a Cronbach's α coefficient of 0.86, a good fit for validity indicators, χ^2^/*df* = 3.64, RMSEA = 0.04, CFI = 0.97, TLI = 0.94, and SRMR = 0.03.

### 2.4 Data analysis

In this study, SPSS 26.0 was applied to perform descriptive statistics, correlation analysis, one-way ANOVA, and reliability analysis of the data, AMOS 26.0 was applied to perform validity test of three scales and validity factor analysis, and finally, Mplus 8.3 was applied to perform the test of measurement invariance, latent profile analysis.

## 3 Results

### 3.1 Common method bias test

The data in this study were obtained from the self-reports of the study participants, which may lead to common method bias, and in order to more accurately count the data, Harman's one-way test was used to test them in this study to ensure the accuracy and reliability of the data. The results showed that the questionnaire of this study extracted three common factors with eigenvalues >1. The explained variance of the unrotated first common factor was 25.31%, which was much smaller than the critical criterion of 40% (Zhou and Long, 2004). Therefore, there is no serious common method bias in this study.

### 3.2 Preliminary analysis

The descriptive statistics of this study showed (Table 2) that the frequency of positive body talk, functionality appreciation, and body image flexibility of Chinese college students was at the medium-high level, while body appreciation was at the medium-low level. The results of correlation analyses showed that body appreciation was significantly and positively correlated with positive body talk, functionality appreciation, and body image flexibility, respectively; positive body talk was significantly and positively correlated with functionality appreciation and body image flexibility, respectively, and functionality appreciation was significantly and positively correlated with body image flexibility.

**Table 2:** Descriptive statistics and correlation analysis of variables (*n* = 625).

**Variable**	***M ±SD***	**1**	**2**	**3**	**4**
Body appreciation	2.98 ± 0.93	1			
Functionality appreciation	3.04 ± 0.95	0.75^**^	1		
Body image flexibility	3.51 ± 1.14	0.26^**^	0.28^**^	1	
Positive body talk	3.11 ± 0.94	0.72^**^	0.61^**^	0.31^**^	1

^**^Indicates *p* < 0.01.

### 3.3 Factor analysis of three measures of positive body image

In this study, the three-factor model of positive body image was subjected to a validated factor analysis in the overall sample, the boys' sample, and the girls' sample. The results showed (Table 3) that the fit indices of the three-factor model of positive body image were met in the overall study sample (*n* = 625), the males' sample (*n* = 399), and the females' sample (*n* = 226), which is a good indication that body appreciation, functionality appreciation, and body image flexibility, respectively, as the three important dimensions of positive body image, are able to measure the overall positive body image of an individual in a good way.

**Table 3:** Results of fitting the three-factor model for positive body image.

	**χ^2^ */df***	**GFI**	**CFI**	**TLI**	**RMSEA (90% *CI*)**	**SRMR**
Overall sample	2.97	0.95	0.96	0.95	0.056 [0.048, 0.064]	0.04
Male sample	2.12	0.94	0.96	0.95	0.053 [0.043, 0.064]	0.03
Female sample	2.26	0.90	0.94	0.93	0.075 [0.061, 0.089]	0.05

### 3.4 Measurement invariance test based on gender

The study used four models (Models 1-4) for multi-group validation factor analysis, and it was found that the CFI and TLI indexes of the morphological invariance, weak invariance, strong invariance, and strict invariance models of the three scales of positive body image ranged from 0.946 to 0.956, the RMSEA and SRMR indexes ranged from 0.053 to 0.060, and the χ^2^/*df* ranged from between 2.01 and 2.14, and the model fit indices were all up to standard, and the results are shown in Table 4. According to the degree of strictness, the measurement invariance of the test scales was classified in order: morphological invariance (configural invariance), unit invariance/weak invariance (metric/weak invariance), scale invariance/strong invariance (scalar/strong invariance), and strict invariance (strict invariance) (Wang et al., 2020). If the fitting indices of the four invariance models are all up to standard, it means that the scale has good measurement invariance (Huang et al., 2023). Through two-by-two comparisons among the four models, the absolute values of ΔCFI and ΔRMSEA are < 0.01. Therefore, it can be well illustrated that the measurement invariance of the three measurement instruments of positive body image on the variable of gender is established.

**Table 4:** Measurement invariance test based on gender variables.

**Model**	**χ^2^**	***df***	**χ^2^*/df***	**CFI**	**TLI**	**RMSEA (90% *CI*)**	**SRMR**	**ΔCFI**	**ΔRMSEA**
Model 1	372.546	174	2.14	0.956	0.946	0.060 [0.052, 0.069]	0.053		
Model 2	390.265	186	2.10	0.954	0.948	0.059 [0.051, 0.068]	0.057	−0.002	−0.001
Model 3	407.144	198	2.06	0.953	0.950	0.058 [0.050, 0.066]	0.057	−0.001	−0.001
Model 4	423.376	210	2.01	0.951	0.952	0.057 [0.049, 0.063]	0.056	−0.002	−0.001

Models 1-4 represent the configural invariance model, weak invariance model, strong invariance model and strict invariance model, respectively.

### 3.5 Latent profile analysis of positive body image

In this study, model fitting was performed for 2–5 categories using 3 variables of body appreciation, functionality appreciation, and body image flexibility as indicators, and model fit parameters were calculated and compared, and the specific fit indices are shown (Table 5). As can be seen in Table 5 below, the values of AIC, BIC, and aBIC kept decreasing with the increase of the categories, i.e., the model's goodness-of-fit improved with the increase of the number of categories. Smaller values for AIC and BIC indicate a better-fitting model. Entropy is an index for predicting the quality of class assignments, and a higher entropy refers to a higher classification precision (Rosenberg et al., 2018). However, the LMRT values are not significant in the 4 and 5-category models. Meanwhile, the percentage of each category in the 3-category is relatively balanced and the Entropy result value of the 3-category model is larger compared to the 2-category model, which indicates that the classification is more accurate and the 3-category is more detailed; therefore, the 3-category is identified as the final latent profile model on a comprehensive basis.

**Table 5:** Latent profile model fitting information.

**Model**	**AIC**	**BIC**	**aBIC**	**Entropy**	**LMRT(*P*)**	**BLRT(*P*)**	**Categorical probability**
2	28432.613	28636.750	28490.706	0.901	< 0.01	< 0.01	0.522/0.478
**3**	**27688.719**	**27963.859**	**27767.018**	**0.902**	**< 0.01**	**< 0.01**	**0.322/0.404/0.274**
4	27364.061	27710.206	27462.567	0.904	0.012	< 0.01	0.315/0.389/0.106/0.190
5	27028.590	27445.739	27147.302	0.907	0.044	< 0.01	0.249/0.096/0.360/0.104/0.191

Bold values represents the categorical model with the best model fitting indices.

The probability of attribution for each profile of the three classifications is shown in Table 6, with the average probability of attribution of positive body image in each profile ranging from 95% to 97%, which suggests that the model of the three classifications is optimal. In this study, the positive body image of Chinese college students was classified into three latent profiles for subsequent analysis.

**Table 6:** Mean probability of attribution in the three classifications.

	**C1**	**C2**	**C3**
C1	0.97	0.03	0
C2	0.03	0.95	0.02
C3	0	0.04	0.96

### 3.6 Differences in positive body image across latent categories

**Figure 1:**
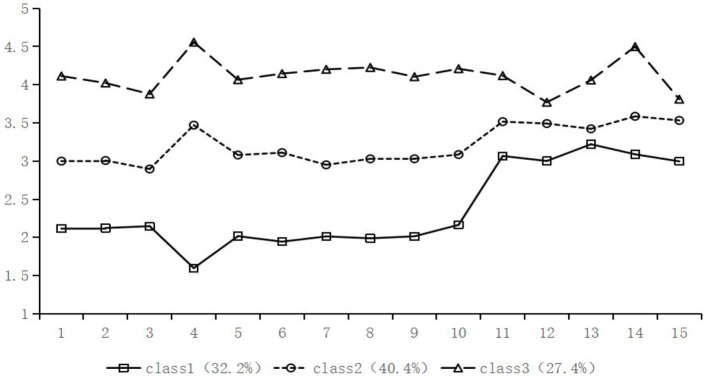
Mean values of items across latent categories of positive body image.

The mean scores of the three potential categories of positive body image for each entry are shown in Figure 1. Category 1 consisted of 200 people (32.2%), which had the lowest scores on all dimensions and was named “low-level positive body image”; category 2 consisted of 253 people (40.4%), which had medium scores on all dimensions and was named “general-level positive body image”; category 3 consisted of 172 people (27.4%), which had the highest scores in all dimensions and was named “high-level positive body image”.

In this study, the subjects were divided into three categories based on the results of the latent profile analyses, and the scores of the Chinese college students on the three dimensions of positive body image under each category were calculated separately (see Table 7). The results of one-way ANOVA showed that there was a significant difference between the different latent categories of positive body image in all three dimensions (*p* < 0.001). Comparisons based on *post-hoc* tests revealed that the scores of the high-level group were significantly higher than those of the remaining two groups on each of the dimensions of Body Appreciation, Functionality Appreciation, and Body Image Flexibility (*ps* < 0.001), with the scores of the three dimensions decreasing sequentially.

**Table 7:** Sub-dimensional comparison of different latent categories of positive body image.

**Dimension**	**Latent categories of positive body image**			**Differential test**	**After-test**
	**Class 1**	**Class 2**	**Class 3**		
Body appreciation	2.12	2.96	4.01	*F* = 475.13^***^	C3 > C2 > C1
Functionality appreciation	1.95	3.10	4.21	*F* = 1,645.25^***^	C3 > C2 > C1
Body image flexibility	3.07	3.49	4.07	*F* = 39.57^***^	C3 > C2 > C1

^***^Indicates *p* < 0.001.

### 3.7 The effect of positive body talk on positive body image

Based on the correlation analysis, this study, in order to further examine the predictive power of positive body talk on the dimensions of positive body image, used multiple linear regression analysis, the results of which are shown in Table 8, with the adjusted coefficients of determination of 0.52, 0.37, and 0.09, which suggests that positive body talk predicts 52% of the variance in body appreciation, functionality appreciation, and body image flexibility, respectively, 37%, and 9% of the variance.

**Table 8:** Multiple regression analysis of positive body talk on dimensions of positive body image.

**Outcome variable**	**Predictor variable**	***R^2^***	**Adjusted *R^2^***	***F***	**β**	***t***
Body appreciation	Positive body talk	0.52	0.52	685.30^***^	0.72	26.17^***^
Functionality appreciation		0.37	0.37	369.36^***^	0.61	19.21^***^
Body image flexibility		0.09	0.09	66.94^***^	0.31	8.18^***^

^***^Indicates *p* < 0.001.

## 4 Discussion

### 4.1 Integration of three measures of positive body image

In this study, the measurement tool of positive body image was integrated and tested in a group of Chinese college students, and the results of a single-group validated factor analysis found that the three-dimensional positive body image measurement tool showed good reliability and goodness of fit in the overall sample, male college student sample, and female college student sample, respectively, which suggests that the three-dimensional positive body image measurement tool is able to measure well the Individuals' overall level of positive body image, which is consistent with the conceptualization of previous research (Yang et al., 2023) and in line with the findings of previous research (Soulliard and Vander Wal, 2022), this reinforces the notion that positive body image is a multidimensional concept consisting of multiple dimensions of body appreciation, functionality appreciation, and body image flexibility as measured by the Body Appreciation Scale-2 (BAS-2), the Functionality Appreciation Scale (FAS), and the Body Image Acceptance and Action Questionnaire-5 (BI-AAQ-5).

### 4.2 Measurement invariance based on gender variable

This study was the first to test the measurement invariance of the three measures of positive body image across genders in a sample of Chinese college students. The results of a multi-group validation factor analysis showed that the four models of configural invariance, weak invariance, strong invariance, and strict invariance of the three measures of positive body image across genders met the psychometric criteria to satisfy measurement invariance across genders. This result suggests that the three measures of positive body image, BAS-2, FAS, and BIAAQ-5, have the same units and reference points between male and female college student populations, that differences in the observed indicators between college students of different genders can be accounted for by the latent variables, and that comparing the scores of positive body image among college students of different genders is measurably meaningful (Chen L. et al., 2023). Therefore, future studies using the three instruments to measure positive body image in college student populations will not be affected by gender, and their measurements truly reflect differences in positive body image, allowing cross-group comparisons across genders (Chen W. et al., 2023; Liu et al., 2024).

### 4.3 Latent categories of positive body image

This study used latent profile analysis to identify three different types of positive body image in a sample of Chinese college students: “low-level positive body image”, “general-level positive body image”, and “high-level positive body image”, and there was continuity in each of the three entries of the three dimensions of positive body image, which is in line with the findings of previous research (Hoffmann and Warschburger, 2018). At the same time, body image flexibility scores were significantly higher than the remaining two dimensions, which suggests that today, under the guidance of the “Healthy China 2030 Program”, social media platforms are full of positive messages and statements such as “I want to love my body”, which inadvertently influences the college population. These positive messages and statements have a strong influence on the body image of college students, and they are able to adjust and improve their concepts, thoughts and behaviors about their bodies according to the positive messages. Objectification theory suggests that women face higher social pressures than men, and that society requires women to evaluate their bodies based on physical appearance rather than on other aspects such as physical function (Fredrickson and Roberts, 1997; Jia and Chen, 2024), which may make it more likely that the female population would be categorized as having a low level of positive body image. However, the present study did not find an effect of gender on the different potential categories of positive body image, and there is currently no evidence to support this result. With the advent of the “national fitness” era, both male and female college students are actively participating in sports activities, which indirectly increases their levels of positive body image (Jiang et al., 2018), so there may not be a gender difference.

### 4.4 The impact of positive body talk on positive body image

It was found that positive body talk was significantly positively related to all three dimensions of positive body image and positively predicted an individual's overall positive body image, which is consistent with the results of previous studies ((He et al., 2023; Alleva and Tylka, 2021). This result suggests that when talking about body-related topics with others in a positive and energizing way (e.g., complimenting and appreciating one's own body), is effective in increasing an individual's overall positive body image, suggesting that positive body talk is a central factor influencing college students' positive body image. Positive body talk is also an important basis for peer selection (Tylka, 2011), and positive body talk with others is more likely to help individuals establish healthy peer relationships, which in turn constructs good mental health. Therefore, one way to promote positive body image among college students is to motivate them to engage in positive body talk with their peers, which colleges and universities can do by conducting group counseling and positive thinking meditation (Alleva et al., 2017).

## 5 Conclusion

Under the conditions of this study, the following important conclusions were drawn: (1) The three measures of positive body image can measure the overall positive body image of college students well and have measurement invariance on the variable of gender. (2) Positive body image was divided into three latent categories: “low-level of positive body image”, including 201 people (32.2%); and “general-level of positive body image”, including 253 people (40.4%); “high-level positive body image”, including 171 people (27.4%). (3) Positive body talk significantly and positively predicted Chinese college students' overall positive body image. This study explored for the first time the multidimensional conceptualization of positive body image in the Chinese context, validated the theory of holistic positive body image proposed by Tylka and Wood-Barcalow (2015a), and also found positive body talk as a significant influencing factor of positive body image, which has a huge impact on future research in positive body image.

To begin with, with the perspective of positive psychology, based on the holistic positive body image theory proposed by Tylka and Wood-Barcalow (2015a), this is the first study integrating the three measures of positive body image, and takes the validation of the multidimensional concept of positive body image for Chinese college students as a starting point, and explores the influencing factor of positive body image, which is of certain reference value for the mental health education of Chinese universities, for example, group counseling and positive thinking meditation could be used to cultivate positive body image of college students. The results of this study are also clinically informative for the treatment of individuals suffering from body image dysfunction, which can be alleviated by boosting their level of positive body image.

Furthermore, this study still has some limitations. Firstly, this study used a cross-sectional study, which made it difficult to explore and validate the dynamic changes in positive body image over time. Based on the broaden-and-build theory of positive emotions proposed by Fredrickson (2001), the field of positive emotions has examined the ascending spiral of positive emotions broadening cognition, positive coping, and interpersonal trust (i.e., expanding people's mindfulness), which in turn builds behavioral flexibility and personal resources, such as positive thoughts, resilience, social closeness, and physical health. Future study needs to combine cross-sectional and longitudinal studies and use latent growth curve modeling (LGCM) to explore the potential development trending of positive body image over time. Secondly, this study used self-report measurement to collect data, which has certain subjective limitations. Future study needs to test and re-validate the multidimensional conceptualization of positive body image with a combination of experimental and questionnaire methods. Thirdly, this study only explored three dimensions of positive body image and has not yet covered other definitions of positive body image by scholars (e.g., broadly conceptualizing beauty, body acceptance and love, and adaptive appearance investment), so future research could include other dimensional variables for a broader and deeper exploration. What's more, this study chose the variable of positive body talk as the influencing factor of positive body image among Chinese college students, and there may be other influencing factors that may have a different impact on positive body image, such as individual traits (personality type), environmental factors (different cultural backgrounds). Future study can further explore other influencing factors that may affect positive body image in order to research the antecedent and consequent mechanisms of positive body image. Lastly, this study used a convenience sampling method to select the college student sample, such a practice reduces the cost of the study, but to a certain extent, it may not be representative of the overall situation of positive body image among Chinese college students. Future study should try to select a more rigorous sampling method to strengthen the representativeness of the findings.

## Data Availability

The raw data supporting the conclusions of this article will be made available by the authors, without undue reservation.
